# The False Dawn of Polygenic Risk Scores for Human Disease Prediction

**DOI:** 10.3390/jpm12081266

**Published:** 2022-07-31

**Authors:** Anthony F. Herzig, Françoise Clerget-Darpoux, Emmanuelle Génin

**Affiliations:** 1 Inserm, Université de Brest, EFS, CHU Brest, UMR 1078, GGB, F-29200 Brest, France; anthony.herzig@inserm.fr; 2 Université Paris Cité, Inserm, Institut Imagine, Laboratoire Embryologie et Génétique des Malformations, F-75015 Paris, France

**Keywords:** polygenic risk score, polygenic additive model, correlation, causation, association

## Abstract

Polygenic risk scores (PRSs) are being constructed for many diseases and are presented today as a promising avenue in the field of human genetics. These scores aim at predicting the risk of developing a disease by leveraging the many genome-wide association studies (GWAS) conducted during the two last decades. Important investments are being made to improve score estimates by increasing GWAS sample sizes, by developing more sophisticated methods, and by proposing different corrections for potential biases. PRSs have entered the market with direct-to-consumer companies proposing to compute them from saliva samples and even recently to help parents select the healthiest embryos. In this paper, we recall how PRSs arose and question the credit they are given by revisiting underlying assumptions in light of the history of human genetics and by comparing them with estimated breeding values (EBVs) used for selection in livestock.

## 1. From the Early Days of Human Genetics to the Birth of Polygenic Risk Scores (PRS)

PRSs were born from the idea that genetic marker information can be useful to predict and prevent complex diseases. This idea is not new. Fifty years ago, the discovery of the human leucocyte antigens (HLAs) and their associations with many diseases led Nobel Prize winner Jean Dausset to declare the following at the Fifth International HLA Workshop:

«*A relatively important positive or negative association of the HL-A determinant with some well-characterized disease would be of the utmost predictive value, allowing the introduction of preventive treatment, and possibly to the eradication of the disease*». [1]

Numerous HLA workshops were subsequently organized, with worldwide collaborations, in order to refine observed associations and to search for their functional basis. These studies were not very successful at predicting disease risk, but they highlighted some important aspects of the genetic architecture of common diseases. First, they showed that there existed some important genetic variability between populations with differences of allele frequency and linkage disequilibrium observed at all geographic scales, between continents but also between countries within a continent, and even within a country [2,3,4]. The important role of our immune system in our biological functioning and the selective response to environmental attacks and large epidemics probably explain this great variability [5]. Second, it was found that the biological mechanisms underlying observed associations are usually very complex. Heterozygous carriers of a given HLA allele can, for example, be more at risk than homozygotes for this allele; e.g., in type 1 diabetes, DR3/DR4 heterozygotes have a higher risk (OR = 16.59; 95% CI, 13.7–20.1) than DR3/DR3 (OR = 6.32; 95% CI, 5.12–7.80) or DR4/DR4 homozygotes (OR = 5.68; 95% CI, 3.91–8.23) (see the meta-analysis in [6]). Interactions were found between different alleles located at different HLA loci. This is, for example, the case in celiac disease, where a large majority of patients carry the two alleles DQA1*0501 and DQB1*0201 that form a heterodimer [7] and the risk is further increased for individuals homozygous for the DQB1*0201 allele [8]. It was also discovered that an allele that confers risk for a disease could be protective against another disease such as where, for example, the DR15 antigen increases the risk of multiple sclerosis (MS), but decreases the risk of type 1 diabetes or rheumatoid arthritis.

Third, for many diseases, it was found that, even if relative risks for carriers compared with non-carriers of HLA antigens are high, these common HLA antigens confer weak absolute risks to their carriers. Consequently, HLA genotyping is not useful to predict those diseases in the general population. This is well illustrated by the association of DR15 and MS. In the French population, DR15 carriers have a relative risk of 4 compared with non-carriers, but the absolute risk for a DR15 carrier of developing MS is only one per thousand. Similarly, for celiac disease, the relative risk of carriers of the DQA1*0501–DQB1*0201 heterodimer compared with non-carriers is very high, around 24 in Europe, but the positive predictive risk conferred by the heterodimer is less than 1%. Consequently, HLA typing is not useful for prediction at the population level. However, it provides information to the clinician to evaluate the risk for the relative of an affected individual [9].

Predominantly, the risk would be calculated according to the mode of transmission of the disease or, when this mode of transmission was not known, according to previously estimated data. For example, the Gail model [10] was proposed to clinicians to compute the risk for a woman to develop breast cancer based on the following factors: age at menarche, age at first live birth, number of previous biopsies, and number of first-degree relatives with breast cancer. However, a large uncertainty was attached to these risk estimates and there was hope that improvements would come from the mapping of the genes involved. An important step in the history of disease risk prediction was indeed the launching of the Human Genome project in the 1980s. Linkage analyses were carried out in large pedigrees with many affected people. More than 1200 genes were identified, often providing knowledge on the protein involved and the deleterious variants [11]. Monogenic sub-entities were also identified in common and complex diseases; for example, some early-onset cases of Alzheimer disease were found to be due to rare deleterious variants in the APP, PSN1, or PSN2 genes [12]. Other examples include the Maturity-onset diabetes of the young (MODY) forms of diabetes [13] or breast cancer due to deleterious variants in the *BRCA1* [14,15] and *BRCA2* [16,17] genes. In families where a deleterious variant segregated, it became possible to determine with certainty family members who had inherited the deleterious variant and family members who had not, and thus to determine who was at high risk. However, even for disease due to deleterious variants, the reliability of prediction depends on the penetrance; i.e., the probability of the disease developing in a variant carrier. Indeed, this penetrance could be much smaller than 100%, as other factors may play a role in disease expression by modulating it and leading to different severity. These factors can be environmental factors or genetic factors that are then often referred to as modifier genes (see [18] for a discussion on modifier genes). In addition, for monogenic sub-entities of common diseases, the interpretation of family history and thus genetic counselling can be complicated by the fact that, in the same family, there may exist some genetic heterogeneity. This difficulty is well illustrated on breast cancer. Given the high risk of breast cancer for women in the general population, in large families where a pathogenic variant of *BRCA1* is segregating, some women may be affected without carrying the variant. Consequently, familial analysis is very sensitive to the phenotype–genotype correspondence [19]. Of special importance are the degrees of relatedness of the family members with cancer, the ages of disease onset, and the ages of family members who do not have the disease [20,21]. Besides, rare variants of *BRCA1* have different likelihoods of pathogenicity [22] and many of them are even of unknown clinical significance [23]. Adding to these difficulties, there is also the possibility that de novo variants or mosaic variants could cause disease, making diagnosis and disease prediction even more difficult (see, for example, [24,25]).

When the HapMap project was launched in 2002, enthusiasm and hope of geneticists were again at a peak. Sets of tag-SNPs that capture most of the common variation on the human genome were defined and used to construct SNP-arrays to perform genome wide association studies (GWASs). Francis Collins, Director of the NIH, predicted that genetic diagnosis would be available within 10 years, and U.S. President Clinton declared on 26 June 2000: “*it would revolutionize the diagnosis, prevention and treatment of most, if not all, human diseases*”. The International Hapmap Consortium shared this optimistic view: “*The HapMap will allow the discovery of sequence variants that affect common disease, will facilitate development of diagnostic tools, and will enhance our ability to choose targets for therapeutic intervention*”. The results of GWAS were, however, not as expected. Associated loci were found to only confer very small increments in risk. However, even if biological interpretations were difficult, there was hope that GWAS results could be used to predict disease risk. Wray et al. [26] proposed an approach to “predict the genetic risk faced by each individual” based on their genotypes at the SNPs found to be associated with the disease in GWAS. This was the birth of polygenic risk scores (PRSs).

## 2. From GWAS to PRS

The principle of combining information at different genetic markers to construct a genetic score was first introduced by animal geneticists to perform marker-assisted selection [27]. This score was later called estimated breeding value (EBV) [28] and is used to estimate offspring performances for quantitative traits of agricultural interest. The principals of EBV are built themselves on the assumption that it is possible to separate the genetic and environmental variance components of a trait. Furthermore, that there are a (typically large) number of genetic variants that each capture a small part of the genetic variance, and the heritability of the trait can thus be recovered by summing across their contributions; this being the polygenic model of inheritance proposed by R.A. Fisher [29]. PRSs were introduced into human genetics by Wray et al. 2007 [26] according to the same principle of summing across the contribution of many different SNPs, but serve a different purpose; that is, predicting the individual risk to develop a disease. Given the relationship between PRS and disease heritability under the model of polygenic architecture, it was shown by Wray et al. 2007 [26] that the predictive power of a PRS is bounded by the heritability, and that is thus important to achieve the maximum disease heritability. Following these results, many geneticists embarked in the quest for the so-called missing heritability [30,31].

PRSs are constructed as a weighted sum of risk allele counts using effect sizes estimated from GWAS as the weights. The value $PRS_{i}$ for individual *i* based on their genotypes at *N* associated SNPs is computed as follows:

(1) $$PRS_{i}=\overset{N}{\underset{k=1}{\sum }}\beta_{k}G_{i,k}$$

where *k* is the effect size for SNP *k* measured in an independent GWAS (or summary statistics obtained from different GWAS for the same disease) and $G_{i,k}$ is the observed genotype of individual *i* at SNP *k* coded as 0, 1, or 2 to represent the number of minor alleles carried by the individual.

In the literature of PRS construction, importance is placed on the choice of the SNPs to include in the model and their weights. In early PRS manifestations, only the SNPs that reached genome-wide significance in the GWAS of interest were included in the model [26,32]. After the results of Yang et al. [33] showed an increase in height heritability estimates when all SNPs rather than only the significant ones were included, it was suggested that PRSs could be improved by keeping all SNPs and applying some shrinkage methods using, for example, LASSO [34] or ridge regression [35,36]. It is also important to account for linkage disequilibrium (LD) between SNPs in order to correctly measure the contribution of the different genomic regions. This could be done either by modelling LD between SNPs to estimate independent genetic effects or by prioritizing, at each locus, SNPs with the smallest GWAS *p*-value (this is referred to as clumping) (for a discussion of these issues, see Choi et al. 2020 [37]).

## 3. The Different Uses of PRS: From Research to Clinics

Once a PRS is constructed with a set of selected SNPs for a given disease, it can be calculated for any individual by obtaining a DNA sample and genotyping the individual for this set of SNPs or using already available SNP-chip genotyping data for the individual. These PRS estimates have been proposed to be used for different purposes (for a review, see [38]). They could be used as a tool to identify individuals that are at high risk of developing a disease in order to offer them preventive treatments or to encourage them to change their behavior. In this case, individuals are ranked based on their PRS for the studied disease and the focus is on individuals in the top percentiles of the PRS distribution (see, for example, [39]).

This approach is also applied to specific sub-populations, for example, to individuals carrying a rare pathogenic variant. PRSs constructed for such a group of carriers have been proposed as a method to explain the observed incomplete penetrance of such mutations (see, for example, [40,41]).

Leveraging the numerous GWAS results available, PRSs have been built for different diseases in an attempt to “bridge the gap between initial discovery efforts and clinical applications” [42]. Standards have been proposed to report PRSs, as it was suggested that the main limitation to the translation of PRSs into clinical care was the heterogeneity in the way these scores were constructed. It was also suggested that PRSs would be improved by collecting association statistics from larger case-control datasets. Indeed, commenting on the progresses made in agricultural yield by the use of EBV, Wray et al. [43] pointed out the differences in family sizes and show how the GWAS sample size is the key factor for maximizing the accuracy of prediction. Besides family sizes, there are also many other important differences between PRS and EBV that can explain why PRSs will not match clinical expectation.

## 4. Erroneous Assumptions of PRS

In this chapter, we explain under which assumptions PRSs are estimated and why, contrarily to the EBV estimates used by animal geneticists, PRSs cannot be used for human disease prediction.

### 4.1. The Polygenic Additive Liability Model of Common Disease Genetic Architecture

A first difference between PRS and EBV is the studied phenotype. PRS are computed for binary phenotypes—affected or non-affected by a given disease—while EBVs usually consider quantitative phenotypes such as milk production. To extend the polygenic model of binary phenotypes, it is assumed that, underlying this binary disease phenotype, there exists an unobserved quantitative trait, called liability, transmitted under Fisher’s polygenic additive model [29] and a threshold over which individuals contract the disease. This polygenic additive liability (PAL) model was proposed 60 years ago to explain the familial segregation of diseases not compatible with monogenic transmission [44].

When using this model, one assumes that the disease has a homogeneous genetic determinism to which small and random effects of environmental factors are added. These are two strong hypotheses that were already discussed in 1965 by Falconer [45], when he showed that the heritability of liability to diabetes strongly decreased with the age of patients. At that time, the different forms of diabetes were not known and diabetes was considered as a single entity. The only information available to support or reject a disease model was the prevalence of the disease in the general population and among relatives of randomly sampled affected individuals from the studied population. Following Falconer’s observations, there were long debates in the scientific community regarding the PAL model and recognition that many alternative models could explain these observations (for a review, see [46]). In the following years, with the development of molecular biology, it was discovered that diabetes is a complex and highly heterogeneous disease with autoimmune forms (with generally early onset and for which the HLA genes play an important and complex role, denoted type 1 diabetes), forms associated with obesity (more often late onset with an important role of diet, denoted type 2 diabetes), as well as monogenic forms that are themselves heterogeneous [47]. It is clear now that the PAL model does not apply to diabetes as a whole and its relevance to type 1 and type 2 diabetes is also very questionable [48]. Diabetes is not an exception. The hypothesis of genetic homogeneity is also not true for most diseases. In breast cancer, for example, there exist different forms of disease and a single genetic model cannot explain them all. Some monogenic forms are due to rare pathogenic variants in different genes. Other forms not explained by high-risk variants are likely to involve different genes and pathways. Indeed, women affected by these forms show different histologies, different gene-expression profiles, and mutational patterns that translate into variable clinical courses and major differences in response to systemic treatments [49]. When pooling all of these different forms together, it is not possible to obtain reliable estimates of genetic effects. Instead, we expect exactly what is observed; that is, very small odds-ratios over many different SNPs. The PAL model is not rejected, whereas we know that it is not a correct model.

Underlying the PAL model is also the assumption that each environmental factor has a small effect that is independent from the effects of the genetic factors. For most traits however, this is clearly not true. The effect of the environment can be important and a given genotype can react differently across different environments [50]. This is well-known from breeders. Changes in the environment can have a dramatic impact on the efficiency of cattle breeding programs and the accuracy of selection [51,52,53]. This occurs, for example, in the case for milk production, with a significant impact of environmental factors such as temperature and humidity and gene–environment interactions [54,55]. The environment cannot be ignored and, as explained by Feldman and Lewontin [56], “*Partitioning of the causes of variation is really illusory. The genetic variance depends on the distribution of environments and the environmental variance depends on the distribution of genotypes*”. In their footsteps, Burt [57] claims that “*the conceptual (biological) model on which heritability studies [and thus also PRS] depend—that of identifiable separate effects of genes* vs. *the environment on phenotype variance—is unsound*”. Gottlieb [58] considers that genes are part of a “*developmental system*” and Moore and Shenk [59] explain that “*contemporary biology has demonstrated that traits are the product of interactions between genetic and non-genetic factors at every point of the development*”. This is well illustrated, for example, in cerebrovascular diseases, with both lifestyle and intrinsic factors playing a role in the development and the severity of neurodegenerative diseases [60]. In the field of livestock selection, the environment effect can be studied and taken into account (see, for example, [55]). In human populations, environments change between geographical location, between cultural groups, and between generations; they cannot be precisely controlled for.

### 4.2. GWAS and Causal Inference

Underneath PRS is the idea that there are individuals with a genetic makeup that predispose them to developing disease and that it is possible to identify such individuals based on their PRS values. It is thus assumed that the different SNPs used to construct the PRS have a direct or indirect (through linkage disequilibrium) effect on the disease itself. The SNPs used in PRS are identified by association tests on the basis of different genotype distributions in cases and controls. It is thus assumed that these observed differences in genotype distributions reflect a true genetic effect. However, this is not true. The most discussed reason is the well-recognized problem of population stratification, but this is not the sole source of associations that do not reflect direct genetic effects. Stratification can be corrected for or averted by matching cases and controls on ancestry, but association may well still reflect the effect of genetic or environmental factors involved not only on the studied trait, but also on other traits associated with the studied trait [61]. This is, for example, the case of many diseases associated with BMI. Among the SNPs associated with these diseases, there are many SNPs that are in fact associated with BMI and not directly with the disease. Such indirect associations are even used by mendelian randomization methods to study the causal impact of BMI on diseases [62], illustrating the implicit acceptance that GWAS associations reflect true genetic effects. In breast cancer GWAS, for example, associations are found with SNPs located in different genes involved in adipocyte differentiation. This does not mean that these genes are directly responsible for breast cancer. It is more likely that this reflects an indirect association owing to fat cells increasing the production of estrogen and, therefore, promoting the growth of cancer cells. Moreover, some associations of SNPs with breast cancer may in fact reflect a difference between cases and controls for alcohol consumption, as alcohol has also been shown to affect estrogen receptors [63]. Matching cases and controls on all environmental risk is, however, not possible, and controls used in GWAS are usually less exposed to environmental risk factors than the general population. Even in a population considered as genetically homogenous, environmental stratification may well exist and PRSs have been shown to be sensitive to changes in variables such as age, sex, and socio-economic status, as shown in recent studies of the U.K. biobank [64]. Therefore, PRSs not only contain information about genetic factors inherited from parents, but also information on clinical and environmental factors. As Janssens [65] rightly pointed out, PRSs do not measure what they are supposed to measure. This fact is totally ignored by those who integrate PRS values in risk models as an observation, which is independent from clinical and environmental factors.

The same problems do not hold when using EBVs to select the best reproducers. Indeed, the genotypes on which the selection is performed are those of the parents, but the trait is measured in the offspring. Thus, contrarily to PRS, we know that the SNPs considered to estimate EBV tag genetic factors transmitted to the offspring. Moreover, the environment is also far more easily and deeply controlled in an agricultural context than it is in humans.

### 4.3. PRS as a Tool to Predict Individual Risk of Disease

Besides its use to classify individuals in a population according to their disease risk, PRS is also described in the litterature as a measure of disease liability; i.e., the probability of developing the disease. This is very different from the use of EBVs at the population scale, where only a ranking is performed to identify the best reproducers—not on their phenotype, but on what they could transmit to their progeny.

Indeed, when we are interested in the transmission of a trait from one generation to the next, the assumption of an additive effect of risk alleles has no impact as only one of the two alleles of the parent is transmitted to the offspring. However, this assumption can have a tremendous impact in disease risk prediction as dominance and epistatic effects cannot be ignored when assessing the absolute risk of developing a disease. This is well illustrated by the example of celiac disease for which the absolute risk is almost zero for those who are not carriers of the HLA DQ2 or HLA DQ8 heterodimer, regardless of their genotypes on the remaining part of the genome [66].

Moreover, if accounting for gene–environment interactions does not seem to improve the discrimination ability of PRS [67], this is no longer true when it comes to evaluating the absolute risk of an individual to develop a disease. The absolute risk of an individual may be high in a given environment and low or even null in another environment. As outlined by Lewis and Vassos [38], “*the risk arising from one’s genes is dynamic, depending on changing factors*”. Some genes may have weak (or even no) marginal effects, but an important effect through their interaction [68,69,70]. Under such “purely epistatic” models, important genes would be missed by association studies [71], and thus their effect cannot be captured by PRS, potentially leading to dramatic underestimation of disease risk.

To predict the individual absolute risk of disease, one needs to know the genotype–phenotype correspondence and this correspondence can be very complex. In breast cancer, for example, the risks of women carrying a deleterious variant differ according to the gene in which the variant is present, but also, for two of the same gene, according to the variant pathogenicity. This pathogenicity can only be assessed by functional assay and is certainly not predicted by PRS values. However, in the BOADICEA risk model, the absolute risk of a woman carrying a rare pathogenic variant is calculated by considering her PRS value as a risk modifier of the variant effect [72]. This is clearly inadequate because, when trying to explain the risk of a woman carrying a rare pathogenic variant, the phenotype of interest is not the disease status (affected/non affected), but instead, we should consider as a phenotype the clinical variability in the population of affected individuals carrying the same variant [18]. In some predictive models, a familial component is even added to PRS as if PRS was capturing a genetic component not inherited from parents (see, for example, [73,74,75]). This is a rather strange interpretation of the PAL model and it is important to recall that this model was proposed to explain recurrence risk in relatives not compatible with monogenic transmission.

Another important misconception is the interpretation of statistics such as the area under the receiver operating characteristic curve (AUC). There is a general confusion between the capacity of PRS to discriminate between affected and unaffected individuals in a population quantified by AUC and PRS predictive power of individual status (affected vs. unaffected). AUC is wrongly considered by many as a precision measure of individual risk and used to measure the clinical utility of PRS [72,75]. However, as shown by Wald and Old [76], PRS may be a very poor predictor of absolute risk in terms of specificity and sensitivity. Taking the example of coronary artery disease, where high AUC are found, Wald and Old [76] show on the data of Khera et al. [77] (AUC = 0.81) and Inouye et al. [78] (AUC = 0.79) that, for a false positive rate of 5%, 85% and 87% of true positives, respectively, would be missed. As explained by Janssen and Martens [79]: “*AUC is a measure of the discriminative ability of prediction models. The assessment of prediction models should be supplemented with other metrics to assess their clinical utility*”. The same questioning on the clinical utility of AUC exists in the field of imaging, where some authors have called for the use of net benefit instead of AUC [80]. It is important to realize that high AUC is not proof of the clinical utility of PRS.

## 5. Discussion

Forgetting the original purpose of GWAS—namely a preliminary step towards understanding the complex and unknown etiology of human diseases—many GWAS now focus on developing PRS. Since its promotion—during the 2007–2010 years—the number of studies devoted to PRS has increased exponentially.

Unfortunately, it is under erroneous assumptions that these scores are computed. As for heritability estimates, PRS depends on an underlying genetic model that is unknown for most human diseases and, in our view, PRS is another resurgence of what we previously described as the GIGO syndrome [81]. The Polygenic Additive Liability model, under which PRS is computed, was proposed sixty years ago to explain the familial segregations of a disease that cannot be explained by a monogenic transmission model. Since then, human geneticists have learned that such a model cannot account for the heterogeneity and complexity of pathophysiological processes. The adoption of this model accredits the idea that our diseases are genetically determined and that our genetic risks of contracting a disease are known at birth.

The misinterpretation of PRS has dangerous clinical and eugenic consequences. Some argue that individuals with a high PRS deserve the same attention than rare mutations carriers and defend the use of PRS in clinical practice [78,82,83]. More and more clinicians are considering using PRS in therapeutic prevention. Furthermore, after years of individual medical future selling by direct-to-consumer companies, new companies are now offering prenatal diagnosis, based on PRS, for all the diseases that parents want to avoid infecting their children [84,85]. Some already protested—from an ethical standpoint—against such eugenic drift [86,87]. However, in addition to the ethical question, one must also be aware that it is scientifically wrong. It is important to stop selling the dream that it is possible to predict disease risk with an over simplistic model. As we have shown, the estimated breeding value is not used in the same way as polygenic risk scores. When using EBVs for selection, phenotypes are measured in the offspring and under well controlled environmental conditions. This may be successful for improving a trait in livestock, but there is no equivalence by which its success might be transferred with the envisioned use of PRS as a universal measure of risk to develop a disease.

Finally, sticking with the single association information, even in very huge samples, is not sufficient to progress in complex disease etiologies. In particular, it is really strange that so many geneticists have turned away from the information provided by familial correlation. There is a huge gap between observing associations in a population and understanding the role of genes in the disease development process [88]. Recent technological advances have offered new possibilities to study the 3D genome organization and shown that chromatin conformation is important to ensure genome function by allowing interactions between different genomic regions. Protein–protein interaction networks have been described that showed that interactions exist at all scales. However, in statistical genetics, we are using a model proposed a century ago when almost no molecular data were available and that ignores all interactions. As already stressed by Nelson et al. [89], it is more than time to move forward and propose alternative models that take better account of the advances made on our understanding of the complex biological processes that drive human phenotypes. Attempts have been made in this direction with, for example, the omnigenic model [90], but it is important to continue on this path and to stop selling the dream that it is possible to predict disease risk with an over simplistic model.
